# Mycorrhizal Inoculation Enhances Nutrient Absorption and Induces Insect-Resistant Defense of *Elymus nutans*

**DOI:** 10.3389/fpls.2022.898969

**Published:** 2022-05-31

**Authors:** Wantong Zhang, Lu Yu, Bing Han, Kesi Liu, Xinqing Shao

**Affiliations:** Department of Grassland Resources and Ecology, College of Grassland Science and Technology, China Agricultural University, Beijing, China

**Keywords:** arbuscular mycorrhizal fungi, *Elymus nutans*, plants defense, volatile organic compounds, *Locusta migratoria*, *Funneliformis mosseae*

## Abstract

The majority of terrestrial plants can form symbiotic associations on their roots with arbuscular mycorrhizal fungi (AMF) in the soil to stimulate the growth and nutrient uptake of the host plant and to improve plant resistance to insects and disease. However, the use of AMF for insect control on gramineous forages requires further study. Here, we evaluated the effects of AMF (*Funneliformis mosseae*) inoculation on the defense against *Locusta migratoria* attack in *Elymus nutans*. Inoculation assays showed that mycorrhizal plants had a higher resistance than non-inoculated plants, as evidenced by plants having more plant biomass, a higher nitrogen and phosphorus content, and greater lipoxygenase (LOX) activity. The results of insect damage showed that in addition to a decrease in the enzyme phenylalanine-ammonia-lyase, the activities of other plant defense-related enzymes (including polyphenol oxidase and β-1,3-glucanase) were increased. A key enzyme, LOX, belonging to the jasmonic acid (JA) signaling pathway was notably increased in mycorrhizal treatment. Volatile organic compounds (VOCs) were identified using gas chromatography mass spectrometry and the results showed that several metabolites with insect-resistant properties, including D-Limonene, p-Xylene, 1,3-Diethylbenzene were detected in mycorrhizal plants. These findings suggest that mycorrhizal inoculation has potential applications in insect management on forage grasses and demonstrates that the JA signaling pathway is essential for insect resistance in *Elymus nutans*.

## Introduction

Plants have evolved a series of complex, protective strategies in order to enhance their fitness and survival under herbivore attack and biotic stresses (Sharma et al., 2017; Frew et al., 2022). One important strategy is the formation of symbiotic relationships between plant roots and some specific fungi, such as arbuscular mycorrhiza (Begum et al., 2019; Dowarah et al., 2021).

Arbuscular mycorrhizal fungi (AMF) belong to the subphylum Glomeromycotina of the phylum Mucoromycota (Spatafora et al., 2016). They are important beneficial fungi that form symbiotic relationships with over 90% of plant roots including ferns, herbaceous plants, and some economically important crop species (Begum et al., 2019; Selvaraj et al., 2020). AMF are obligate biotrophs that ingest plant photosynthetic products (Dowarah et al., 2021) and lipids to support their life cycle (Jiang et al., 2017). AMF symbiosis can help plants to obtain essential nutrients from the soil, such as nitrogen, phosphorus, and potassium, absorb other trace nutrients from resource-deficient soils, and assist plant roots to take-up more water from the soil (Kayama and Yamanaka, 2014; Bhantana et al., 2021; Jiang et al., 2021). In return, plants supply AMF with carbohydrates and lipids that can be used to develop extensive mycelial networks (Bernaola et al., 2018). These mycelial networks act as conduits for carbon and mineral nutrient transmission (Jiang et al., 2017; Luginbuehl et al., 2017); they also signal transmission channels between plants to initiate an early warning for disease and herbivore attack (Basu et al., 2018; Begum et al., 2019).

Arbuscular mycorrhizal fungi confer benefits to their host plant in many ways, including enhancement in nutrient uptake and protection against stressors (Dowarah et al., 2021). Most importantly, AMF inoculation is known to induce the activation of plant resistance and protect plants from phytopathogenic fungi, bacteria, viruses, and herbivores (Hao et al., 2019; Miozzi et al., 2019; Jiang et al., 2021). Mycorrhizal symbiosis have been reported to respond variably to above-ground insect damage, including positive (Babikova et al., 2013; Meier and Hunter, 2018), negative (Bernaola et al., 2018; Bolin et al., 2018), and neutral (Gehring and Bennett, 2009) effects on plant defense. In addition, the defense response of mycorrhizal plants to herbivorous insects has been found to vary with the species involved and the growth stage of the plant (Malik et al., 2018) and the AMF (Selvaraj et al., 2020). Specifically, mycorrhizal inoculation improves plant resistance to generalist herbivores and insects that are sensitive to jasmonic acid (JA)-associated defenses (Frew et al., 2022).

Plants can release distinct blends of volatile organic compounds (VOCs) following an external attack, including alkenes, terpenes, and aldehydes (Sharma et al., 2017; Ye et al., 2018). VOCs can be classified according to their metabolic pathway: terpenoids [mevalonate/2-methyl-D-erythritol-4-phosphate (MVA/MEP) pathway], volatile fatty acid derivatives [lipoxygenase (LOX) pathway], and benzenic compounds and amino acid derivatives [shikimate (SK) pathway] (Dudareva et al., 2004; Velásquez et al., 2020a). Plants can communicate with both distant plants (Heil and Ton, 2008), and neighboring plants (Karban et al., 2016) *via* VOCs, and this airborne signal allows receptor plants to invoke defense systems prior to the threat. Specifically, VOCs can induce the expression and production of defense-related enzymes (polyphenol oxidase, PPO; β-1,3-glucanase, phenylalanine-ammonia-lyase, PAL; and lipoxygenase, LOX), hormones (JA, and salicylic acid, SA), protease inhibitor I and II genes (PI-I and PI-II) and other related secondary metabolites in recipient plants, activating the defense system (Jung et al., 2012; Song et al., 2013). Recent studies have found that plant volatile emissions are strongly influenced by environmental stimuli, including plant-microorganism interaction which plays a key role in plant development (Valenzuela et al., 2019; Velásquez et al., 2020a). For one thing, the impact of mycorrhizal symbiosis extends beyond plant growth, enhancing plant defenses by promoting the synthesis of secondary metabolites such as VOC. For example, milkweeds inoculated with AMF release more VOC after being fed on by insects (Meier and Hunter, 2019). For another, AMF affect the diversity of plant primary and secondary metabolites by altering plant nutrient uptake and defensive signaling pathways (Schweiger et al., 2014; Begum et al., 2019), including the SA and JA pathways (Frew et al., 2022), which are essential defense systems to herbivore feeding (Meier and Hunter, 2019; Dowarah et al., 2021). Song Y. Y. et al. (2015) showed that JA-related genes and defense enzymes (LOX and PPO) are notably up-regulated in receptor tomato plants through common mycorrhizal networks, when neighboring plants are eaten by herbivores. However, these mycorrhiza-related studies mainly focused on cultivated crops (Babikova et al., 2013; Song Y. Y. et al., 2015; Selvaraj et al., 2020) and less information is available regarding the tripartite interactions among AM fungi, gramineous forage, and phytophagous insects.

*Elymus nutans* (*Elymus nutans* Griseb.) is a perennial gramineous grass that grows in alpine regions (Xu et al., 2018). It is highly resistant to drought, cold, and salt, and has become a preferred forage species for planting in high altitude areas (Tan et al., 2020; Quan et al., 2021). As the forage livestock industry expands at high altitudes, the areas of cultivated grasslands growing *E. nutans* will continue to increase; therefore, the stability of *E. nutans* swards in alpine regions needs to be addressed. Grasshopper feeding can cause necrotic spots or even plant mortality, thus reducing the palatability of *E. nutans* and affecting livestock grazing (Hao H. et al., 2019). Furthermore, *E. nutans* can form a symbiotic relationship with AMF (Gai et al., 2009). However, whether this symbiosis enhances *E. nutans* resistance to grasshopper attack and what types of signaling substances are induced by AMF inoculation with *Elymus* spp., transduction pathways, physiological effects, and mechanisms of action have not been systematically explored. Therefore, we selected *E. nutans* in an alpine meadow as host plants to investigate the response of inoculation with *F. mosseae* to insect attack. We sought to (i) determine the response and main defense pathways of *E. nutans* to grasshopper feeding and (ii) elucidate the role of AMF on plant growth and defense against insects. The hypotheses were (i) *E. nutans* will produce large amounts of defense-related enzymes to activate relevant defense pathways. (ii) AMF-colonized plants will have higher nutrient content, increased defense enzyme activity, and will release more VOCs.

## Materials and Methods

### Plant, Fungal, and Insect Materials

*Elymus nutans* seeds were collected from natural alpine meadows in Haiyan County, Haibei Tibetan Autonomous Prefecture, Qinghai Province, China (36°55′ N, 100°57′ E, 3029 m a.s.l.) in August 2021.

The mycorrhizal fungus *Funneliformis mosseae* (BGCYN05), isolated from white clover and maize, was obtained from the Beijing Academy of Agricultural and Forestry Sciences and propagated by the Guizhou Academy of Agricultural Sciences. These AMF inocula contained root fragments, rhizosphere soil, and 133 spores per gram of dry soil.

The grasshoppers *Locusta migratoria* (Orthoptera, Locusta Linnaeus) were collected from a Qinghai Haibei alpine meadow. As the dominant locust species, the density of *L. migratoria* was 10 grasshopper/m^2^ (Hao et al., 2019). They were reared on gramineous plants in the laboratory (28 ± 2°C; 18 h day:8 h night; 30–50% relative humidity) (Babikova et al., 2013; Veenstra et al., 2021).

### Growth Medium

Field soil was obtained from natural alpine grassland in Haiyan County, Haibei Tibetan Autonomous Prefecture, Qinghai Province, China (36°55′ N, 100°57′ E, 3029 m a.s.l.). All soils were sieved with a 2-mm sieve, autoclaved at 121°C for 1 h twice within 3 days and dried at 110°C for 36 h (Li et al., 2021). The physical and chemical properties were: 4.1 g⋅kg^–1^ total nitrogen, 48 g⋅kg^–1^ total carbon, 0.69 g⋅kg^–1^ total phosphorus, and pH 8.2. Each pot was filled with 2.5 kg of sterilized soil.

### Experimental Design

The pot trials took place in a greenhouse at the China Agricultural University from December 2020 to April 2021. *F. mosseae* was used for mycorrhizal inoculation of *E. nutans*, and the grasshopper *L. migratoria* was used for insect feeding. Experiment designs consisted of one AMF inoculation factor (inoculate with *F. mosseae*, F), one insect feeding factor (attack by grasshopper *L. migratoria*, L) and their interaction. The whole experiment had four treatments with five replications each to exclude possible effects of sole AMF inoculation and grasshopper feeding; CK (plants without AMF inoculation and grasshopper feeding), Fm (plants only inoculated with *F. mosseae*), Lm (non-inoculated plants with grasshopper feeding), and Fm + Lm (plants inoculated with AMF plus grasshopper feeding). There were 20 experimental pots, and each pot was placed randomly.

Seeds were surface sterilized with 10% H_2_O_2_ and rinsed five times with sterile distilled water before sowing in autoclaved soil (121°C; 2 h). Forty to fifty plants from the original 80 were chosen randomly per pot and watered regularly four times a week to maintain soil moisture. One week after germination, 40 healthy seedlings with good growth were kept.

Thirty grams of AMF inoculum (fungal: soil mixture) were spread on the soil to a depth of 2 cm. For non-AMF treatments, an equal amount of soil containing autoclaved fungi was added (121°C; 2 h). Insect feeding treatments took place when *E. nutans* plants were 14 weeks old. Before the formal experiment, all plants were placed in polyethylene terephthalate (PET) bags to prevent plant-to-plant communication *via* aerial volatiles, and grasshoppers were starved for 2 h. Subsequently, 12 fifth-instar grasshoppers were placed in each pot of Lm and Fm + Lm treatments to feed on the plants.

After 24 h of grasshopper feeding, we collected VOCs and removed the insects (all grasshoppers were alive). The plants continued growing for 48 h and were then harvested for the measurement of plant nutrient content, biomass, and AMF colonization of roots.

### Mycorrhizal Colonization Analysis

Mycorrhizal colonization was examined following the procedure of Koske and Gemma (1989). After harvesting of plant roots, they were washed with distilled water to remove soil particles, and approximately 0.5–1.0 g of roots were cut into 1 cm segments. Root segments were soaked in KOH (10%, w/v) and boiled until the roots were transparent. Then root segments were acidified in HCl (2%, v/v), and stained with trypan blue (0.05%, w/v). After 30 min, these roots were immersed in destaining solution (glycerol: lactic = 1:1). Finally, stained roots were mounted on slides and AMF colonization was calculated using the magnified gridline intersect method (McGonigle et al., 1990) with a compound microscope under 40× magnification. A root intersection was considered colonized if hyphae, arbuscules, or vesicles were present.

### Enzyme Assays

The kits for assaying the PPO, PAL, β-1,3-glucanase, and LOX were obtained from Solarbio Science & Technology Co., Ltd. (Beijing, China). All the chemicals used were analytical (Li et al., 2019). Leaf samples (0.1 g) were ground in liquid nitrogen with different 1 ml extractive solutions; they were then centrifuged and homogenated, and the supernatants were used for enzyme assays. A Microplate reader (SpectraMax iD5, Molecular Devices, San Jose, CA, United States) and 96-well plates were used. PPO enzyme absorbance was recorded at 410 nm for the measurement and control tubes; a change in absorbance at 410 nm of 0.005/min was defined as a unit of enzyme activity. PAL enzyme absorbance was read at 290 nm; a change in absorbance at 290 nm of 0.05/min was defined as a unit of enzyme activity. β-1,3-glucanase enzyme was measured at 540 nm for the measurement and control tubes, then a standard curve was built to calculate enzyme activity. LOX enzyme catalyzed the oxidation of linolenic acid, and the oxidation product had a characteristic absorption peak at 234 nm; a change in absorbance at 234 nm of 0.0006/min was defined as a unit of enzyme activity.

### Collection of Plant Volatile Organic Compounds

Plants and grasshoppers were placed in PET bags (100 cm × 111 cm, low volatility at high temperatures and high light intensities). After 24 h of insect feeding, volatiles were collected using an aerated kit (Babikova et al., 2013). The air was purified by activated carbon through a dry glass sorbent tube (0.5 cm diameter, 8.0 cm long) and the ends of this tube were plugged with clean glass fiber, containing 50 mg of sorbent (Porapak Q, 80–100 mesh, Waters Corporation, Ireland). After the air had been extracted from the bag for a short time, sampling began after 30 min. During this process, the gas in the bag was adsorbed by the sorbent. The flow rate was 300 ml min^–1^ and the extraction was continuous for 6 h. The sorbent was eluted with 4 ml of chromatographic n-hexane into 2 ml sample bottles and the samples were then stored at −20 °C in a deep freezer for later use.

*GC-MS Analysis of Plant VOCs* We analyzed a 2 μl aliquot of a VOC sample by gas chromatography mass spectrometry (GC-MS, Agilent Technologies, Santa Clara, CA, United States) using the following GC method: Injector maintained at 220°C, initial column temperature maintained at 50°C for 10 min, ramped to 200°C at 5°C min^–1^, and held for 10 min, with a helium carrier gas flow rate of 1 ml min^–1^. We used a DB-5MS column (30 m × 0.25 mm × 0.25 μm film; J&W Scientific, Folsom, CA, United States) with a 0.25-μm film thickness (Velásquez et al., 2020b). Then VOCs were initially identified by using the mass spectra with a library of authentic standards or databases (NIST 08, National Institute of Standards and Technology, Gaithersburg, MD, United States, 2008). The relative concentrations of VOCs were determined by comparing each peak area with the total peak area in each treatment.

We selected random harvested plant material (roots, stems, and leaves) to measure nutrient content. They were dried at 65 °C for 24 h and milled with a ball mill (Retsch MM400, Retsch, Haan, Germany) Samples of 0.15-g sieved plant material were weighed and placed into tin cups. Plant total nitrogen and total carbon contents were determined using an elemental analyzer (Elementar, Hanau, Germany); total phosphorus was determined by the HClO_4_-H_2_SO_4_ method (Han et al., 2021).

### Statistical Analysis

All available data were analyzed by SPSS 19.0 (Inc., Armonk, NY, United States). Two-way analysis of variance (ANOVA) was used to examine the effects of inoculation and insect feeding on plant biomass, defense-related enzyme activities and plant nutrient content. Differences among treatments were performed at *P* < 0.05 by Duncan’s multiple range test (DMRT). The results were given as means with standard errors (mean ± SE). Origin2018 (OriginLab, United States) was used for plotting.

## Results

### Mycorrhizal Colonization Rate and Plant Biomass

**FIGURE 1:**
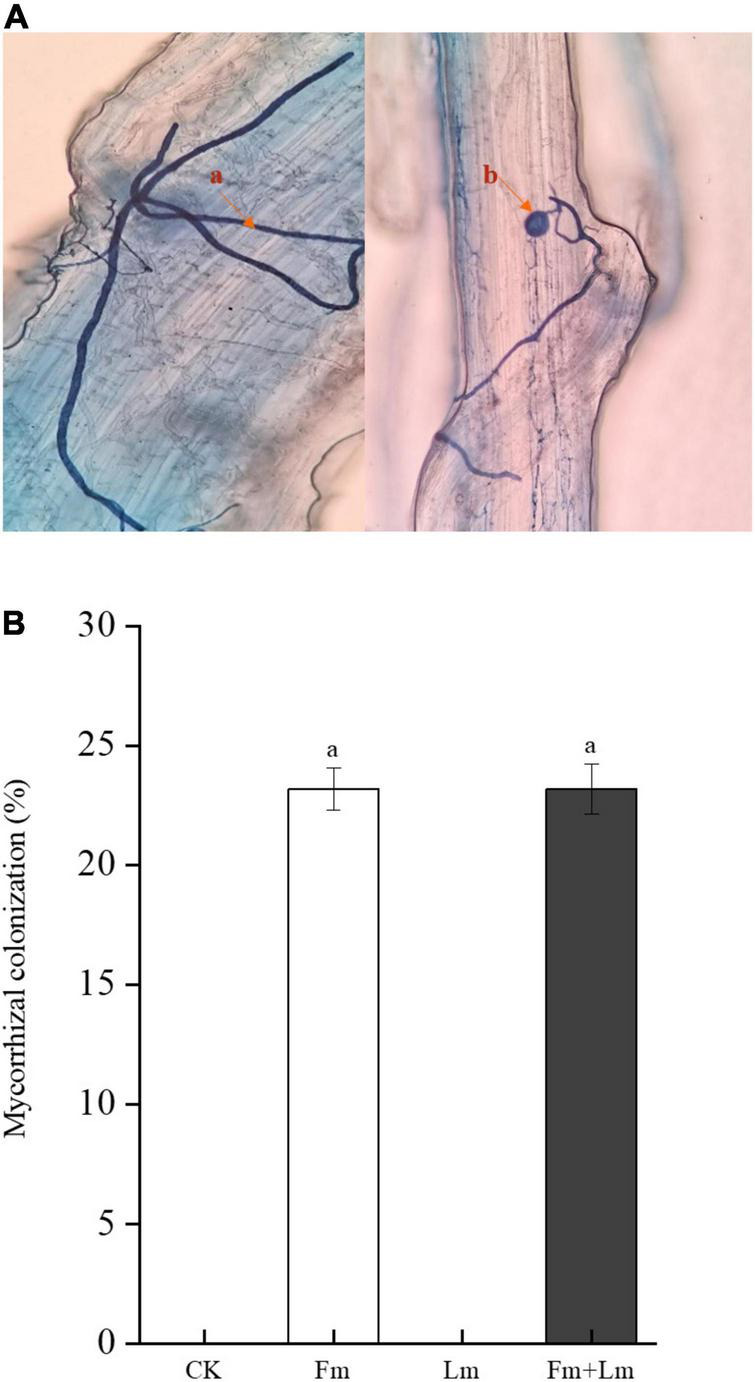
The mycorrhizal colonization structure of AMF observed under 40× magnification after **(A)** the root segments of *E. nutans* were stained with trypan blue **(B)** (0.05%, w/v) and the percentage of root mycorrhizal colonization in plants. The four treatments were (1) CK: no inoculation; (2) Fm: inoculated with *F. mosseae* only; (3) Lm: feeding with *L. migratoria* only; (4) Fm + Lm: inoculated with *F. mosseae* and *L. migratoria* feeding. (a) Internal hyphae; (b) Vesicles.

**FIGURE 2:**
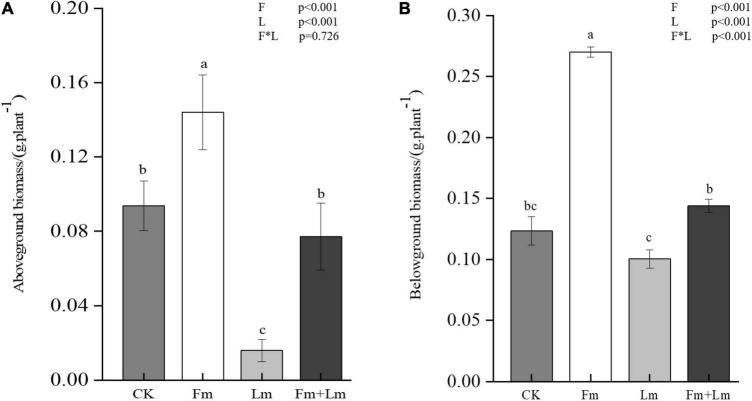
Plant aboveground biomass **(A)** and belowground biomass **(B)** under different treatments. The four treatments were (1) CK: no inoculation; (2) Fm: inoculated with *F. mosseae* only; (3) Lm: feeding with *L. migratoria* only; (4) Fm + Lm: inoculated with *F. mosseae* and *L. migratoria* feeding. Values are means ± standard error. *P*-values for inoculation and insect feeding effects were based on two-way ANOVA, F: inoculated AMF; L: insect feeding. Mean values followed by the same letter do not differ significantly at *P* ≤ *0.05* by DMRT.

Mycorrhizal colonization structures such as vesicles and mycelium were observed in all inoculation treatments, which indicated successful inoculation with AMF (Figure 1A). Mycorrhizal colonization rates were similar between the two inoculation treatments (Figure 1B). Grasshopper feeding resulted in a small amount of aboveground biomass (Supplementary Figure 1). Mycorrhizal inoculation and insect feeding significantly affected plant biomass (*P* < 0.05). The Fm treatment had the greatest aboveground biomass, 53.53% higher than non-inoculated CK treatment (*F* = 13.82; *P* < 0.05). Insect attack caused a decrease in the aboveground biomass of plants, the Fm + Lm treatment was still higher than that of the Lm treatment, similar to the aboveground biomass of plants that were not inoculated (Figure 2A). The belowground biomass of Fm treatment was significantly higher than other treatments (Figure 2B). After the plants were attacked by insects, inoculated plants showed higher belowground biomass, at 43.46% higher in Fm + Lm than in the Lm treatment (*F* = 96.64; *P* < 0.05).

### Plant Nutrient Content

**FIGURE 3:**
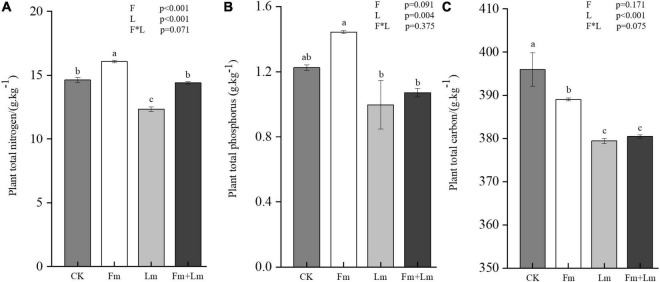
The effects of different treatments on plant nutrition. Total nitrogen, **(A)** total phosphorus, **(B)** and total carbon **(C)** of plant leaves. Four treatments (CK, Fm, Lm, and Fm + Lm) were established as described in Figure 2. Values are means ± standard error. *P*-values for inoculation and insect feeding effects were based on two-way ANOVA. Mean values followed by the same letter do not differ significantly at *P* ≤ 0.05 by DMRT.

Arbuscular mycorrhizal fungi inoculation promoted the uptake of nutrients by *E. nutans*, the inoculated plants contained more nitrogen and phosphorus than non-inoculated plants (Supplementary Table 1). Total nitrogen content in Fm plants was 16.07 g⋅kg^–1^ (Figure 3A), a significant increase of 30.11% compared to CK (*F* = 102.42; *P* < 0.05); But, insect attack significantly affected plant nutrient content (*P* < 0.001). After grasshopper feeding, all insect treatments showed a reduced nutrient content (Figure 3B and Supplementary Table 1). Plant total phosphorus content decreased by 26.70% in Fm + Lm and 18.57% in Lm compared to Fm and CK treatments, respectively. Plant carbon content decreased after AMF inoculation, the total carbon content of CK treatment was 395.98 g⋅kg^–1^, which was notably higher than that of Fm (Figure 3C). Insect feeding further reduced the total carbon content of plants; Fm + Lm decreased by 2.22% compared to Fm and Lm treatments, which decreased by 4.17% (*F* = 15.932; *P* < 0.05).

### Induction of Defense-Related Enzymes by Mycorrhizal Inoculation

Both inoculation and insect feeding significantly influenced the expression of plant defense-related enzyme activities (*P* < 0.05) (Table 1). PPO and β-1,3-glucanase enzyme activities were significantly lower in the Fm treatment compared to the CK treatment (*P* < 0.05). The two enzyme activities were increased in Fm + Lm and Lm treatments after insect attack, with 65.79 and 19.83% increase in Lm treatment compared to CK treatment, respectively. However, mycorrhizal inoculation decreased the expression of the two enzyme activities in insect treatments (Fm + Lm, Lm), PPO, β-1,3-glucanase enzyme activities in the Fm + Lm treatment were decreased by 54.38 and 17.92% compared to the Lm treatment, respectively. No matter inoculation or insect attack, PAL enzyme activity was decreased, but there was no significant differences among treatments.

**TABLE 1:** The activity of four defense-related enzymes in leaves of *Elymus nutans* plants in response to mycorrhizal colonization by *F. mosseae* and herbivorous feeding by fifth-instar grasshoppers *L. migratoria*.

Treatment	PPO (U/g FW)		PAL (U/g FW)		β-1,3-Glucanase (U/g FW)		LOX (U/g FW)	
CK	74.84 ± 0.35b		42.69 ± 7.32a		18.86 ± 0.77ab		481.08 ± 97.12c	
Fm	11.25 ± 2.07d		27.54 ± 4.33a		14.34 ± 0.77c		1846.54 ± 324.47b	
Lm	124.08 ± 0.44a		26.17 ± 5.39a		22.60 ± 2.60a		1277.18 ± 2.83bc	
Fm + Lm	56.60 ± 3.24c		25.02 ± 3.36a		18.55 ± 1.52ab		2903.68 ± 396.47a	

**Variables**	***F***	***P***	***F***	***P***	***F***	***P***	***F***	***P***

F	1137.507	0.000	2.355	0.163	7.138	0.028	32.922	0.000
L	592.560	0.000	3.217	0.111	6.134	0.038	12.631	0.007
F*L	0.998	0.347	1.742	0.223	0.022	0.887	0.251	0.630

*We employed two-way ANOVA to determine the effect of inoculated AMF (F) and insect feeding (L) on four defense-related enzymes. Means with different letters (a, b, c, d) indicate significant differences among four treatments by using DMRT at P ≤ 0.05 level. Five replications per treatment. Values are means ± standard error. (1) CK: non-inoculate plants; (2) Fm: plants inoculated with F. mosseae only; (3) Lm: non-inoculate plants eaten by L. migratoria; (4) Fm + Lm: plants inoculated with F. mosseae and L. migratoria feeding.*

After plants were inoculated with AMF, the LOX enzyme activity of mycorrhizal plants was significantly higher compared to CK treatment (Table 1). And grasshopper feeding caused an increase in LOX enzyme activity in the Lm treatment, which was about 2.5 times higher than that in CK treatment, but still lower than that in the Fm treatment. Moreover, inoculation further increased the expression of LOX enzyme activity in plants, and the LOX enzyme activity in the Fm + Lm treatment was significantly increased by 57.25% (*F* = 15.268; *P* < 0.01) compared with Fm treatment, which was about twice as much as that in the Lm treatment. Two-way ANOVA showed no significant effects of the interaction between inoculation and insect feeding on LOX enzyme activity expression.

### Composition and Concentration of Volatile Organic Compounds

Grasshopper feeding resulted in VOCs released from *E. nutans*. A total of 24 VOCs were produced after plants were attacked by insect, and these metabolites include ketones, aldehydes and benzenic compounds (Table 2). The Lm treatment detected 18 VOCs, of which were “benzenic compounds” (6) (1-methylpropyl Benzene, p-ethyl-Cumene, 4-Ethyltoluene, 1,2,3-Trimethylbenzene, 1,4-Diethylbenzene, Cumene), “ketones” (4) (2′-Methylacetophenone, 2,4-Dimethylacetophenone, 2,5-Dimethylacetophenone, 4′-Ethylacetophenone), “alkenes” (2), “alkanes” (2), “aldehydes” (2), “alcohols” (1), and ethyl acetate. The VOCs with high relative concentrations in the Lm treatment were 2,4-Dimethylacetophenone, β-Cymene, and 1,4-Diethylbenzene. Ethyl acetate was only released from non-AMF plants after grasshopper feeding. There were 19 VOCs detected in Fm + Lm treatment, including “benzenic compounds” (9), “ketones” (3), “alkenes” (3), “alkanes” (2), “aldehydes” (1), “alcohols” (1). Mycorrhizal inoculation led plants to release some different, insect-resistant compounds, like 1,3-Diethylbenzene, D-Limonene, p-Xylene, p-Methylcumene. The VOCs with high relative concentrations in the Fm + Lm treatment were 2,4-dimethylacetophenone, D-Limonene and p-Xylene. Regardless of whether AMF was inoculated or not, 13 common VOCs were detected in *E. nutans* after the insect feeding and the relative concentration of 2,4-dimethylacetophenone was the greatest; however, mycorrhiza slightly reduced emissions.

**TABLE 2:** The composition and concentration of volatile organic compounds (VOCs) collected from plants through GC-MS analysis, included four treatments.

Compound	Relative concentration (%)				CAS
	
	CK	Fm	Lm	Fm + Lm	
Cumene	—	—	0.71 ± 0.32	0.90 ± 0.40	98-82-8
1-methylpropyl Benzene	—	—	0.52 ± 0.23	0.47 ± 0.21	135-98-8
Hydrindene	—	—	—	0.41 ± 0.18	496-11-7
p-Methylcumene	—	—	—	6.51 ± 1.78	99-87-6
para-Ethylstyrene	—	—	1.10 ± 0.22	0.72 ± 0.24	3454-07-07
p-ethyl-Cumene	—	—	1.16 ± 0.34	1.54 ± 0.42	4218-48-8
Ethyl-benzaldehyde	—	—	0.94 ± 0.26	1.32 ± 0.24	4748-78-1
4′-Ethylacetophenone	—	—	2.13 ± 0.95	5.46 ± 2.44	937-30-4
2,4-Dimethylacetophenone	—	—	36.4 ± 4.97	30.08 ± 4.44	89-74-7
4-Ethyltoluene	—	—	2.12 ± 0.58	2.22 ± 0.48	622-96-8
p-Propyltoluene	—	—	—	0.45 ± 0.20	1074-55-1
β-Cymene	—	—	11.55 ± 1.50	4.37 ± 1.43	535-77-3
Isobutenylbenzene	—	—	0.50 ± 0.22	0.54 ± 0.247	768-49-0
2,5-Dimethylacetophenone	—	—	10.72 ± 3.32	5.64 ± 2.52	2142-73-6
1,3-Diethylbenzene	—	—	—	6.44 ± 2.88	141-93-5
D-Limonene	—	—	—	6.90 ± 3.09	7705-14-8
p-Xylene	—	—	—	6.66 ± 2.98	106-42-3
Phenethyl alcohol	—	—	0.96 ± 0.26	0.41 ± 0.19	1123-85-9
2-methylcyclopropyl benzene	—	—	0.53 ± 0.24	0.52 ± 0.23	3145-76-4
2-Phenyl-1-propanal	—	—	0.49 ± 0.22	—	93-53-8
ethyl acetate	—	—	9.86 ± 4.40		141-78-6
1,2,3-Trimethylbenzene	—	—	0.39 ± 0.17	—	526-73-8
1,4-Diethylbenzene	—	—	15.26 ± 6.82	—	105-05-5
2′-Methylacetophenone	—	—	0.31 ± 0.14	—	577-16-2

*Five replications per treatment. Data are expressed as mean ± standard deviation. (1) CK (non-inoculate plants); (2) Fm: (plants inoculated with F. mosseae only); (3) Lm (non-inoculated plants with L. migratoria feeding); (4) Fm + Lm (plants with inoculated F. mosseae plus L. migratoria feeding); (3) CAS (Chemical abstracts service).*

## Discussion

Mycorrhizal symbiosis as a potential pest control strategy may be an effective alternative to some chemical pesticides in contemporary agriculture (Jiang et al., 2021). However, the applicability of this insect resistance mechanism in alpine forage plants is unknown. In the present study, we investigated the results of insect–plant–microbe interactions, the first report of a tripartite interaction between *Elymus nutans*, AMF, and a chewing insect.

It has been commonly reported in both laboratory and field experiments that plant inoculation with AMF promote growth and increase biomass, a phenomenon considered as the positive mycorrhizal growth response (MGR) (Bernaola et al., 2018; Zhang et al., 2019). Positive MGR was previously found to be due to the improved uptake and transfer of nutrients (usually phosphorus and nitrogen) (Jiang et al., 2021), which is consistent with the findings of this study. *E. nutans* inoculated with *F. mosseae* significantly increased plant biomass compared to the non-inoculated treatment, in addition to increasing the uptake of nitrogen and phosphorus. This improvement is probably because a common mycorrhizal network can greatly improve the efficiency of use of soil nutrients by increasing the contact area between the root system and soil *via* a greater number of extraradical hyphae (Begum et al., 2019). There is a consequent increase in concentration of various macro-nutrients and micro-nutrients in plants, which increases the production of photosynthetic products, and leads to greater biomass accumulation (Balliu et al., 2015; Chen et al., 2017). Therefore, AMF assist plant development under normal as well as stressful circumstances and improve plant tolerance to biotic and abiotic factors (Plassard and Dell, 2010; Begum et al., 2019).

The soil used in this study was native soil from alpine meadows where the *F. mosseae* had a good symbiotic relationship with *E. nutans* (Gai et al., 2009), but we used commercial AMF inocula whose host plants were maize and clover, the experiment results revealed a low mycorrhizal colonization rate (Jin et al., 2011; Xu et al., 2018). The reason may be explained by the fact when *E. nutans* inoculated with AMF from the other plants rhizosphere soil, the other plants produced secondary metabolites which cause negative effects on plant growth (Wang et al., 2019), and possibly because of the lignification of plant roots intensified—it was not favorable for AMF colonization (Xu et al., 2018). However, AMF could still promote the nutrient uptake and utilization of *E. nutans*, probably because the small amount of AMF inocula diluted the potential allelopathy effects of the host plant (Wang et al., 2019). As Bati et al. (2015) showed, irrespective of mycorrhizal inoculation (commercial or native), AMF promoted nutrient uptake and plant growth. Therefore, the low colonization rate of *E. nutans* in the current experiment was acceptable. The total carbon in plants is an important physiological parameter reflecting their carbon metabolism and an important indicator of a plant’s physiological condition, growth, vitality, and disease resistance (Jiang et al., 2017; Charters et al., 2020). Extra disturbance will affect the carbon allocation of plants and could then influence AMF activity (Walder and van der Heijden, 2015; Charters et al., 2020). We found that after AMF inoculation, the total carbon content of plants (Fm, Fm + Lm) decreased compared with the non-inoculated treatment (CK), while AMF inoculation (Fm) increased the phosphorus content of plants. However, after grasshopper feeding, the total carbon and phosphorus content decreased in the Fm + Lm treatment. These results suggested that the insects reduced belowground plant carbon allocation and ultimately aboveground phosphorus, possibly as AMF resulted in a lower transfer of phosphorus to the host plant (Frew, 2021). Girousse et al. (2005) found that aphid feeding had a profound effect on plant carbon allocation, which was detrimental to mycorrhizal fungi. The result indicated that herbivore insects could drive asymmetry in the nutrient exchange between mycorrhizal symbionts (Walder and van der Heijden, 2015). We therefore speculated that short-term changes in external biological carbon sinks may have altered the nutrient supply of *E. nutans* to AMF; this finding was similar to that of Charters et al. (2020), but there was insufficient evidence to prove this. Greater attention could be paid to this point in future experiments.

Mycorrhizal inoculation enhances direct and indirect plant defense systems (Jung et al., 2012; Velásquez et al., 2020b). When inoculated plants are eaten by herbivores, mycorrhizal symbionts increase the release of defense metabolites, through interactions with phytohormone signaling pathways (Meier and Hunter, 2018). Study have demonstrated that the JA signaling pathway plays a critical role in mediating plant defense in response to herbivorous insects (Frew et al., 2022). Therefore, we tested defense-related enzyme activities including PPO, which catalyzes the formation of lignin and other oxidative phenols (Song et al., 2010). PAL is involved in the biosynthesis of SA signal molecules and phytoalexin or phenolic compounds (Selvaraj et al., 2020). LOX, a key enzyme of the JA signaling pathway, catalyzes the initial reaction in the JA biosynthesis pathway, and β-1,3-glucanase is able to degrade fungal cell walls, causing lysis of fungal cells to participate in the SA defense pathway (Zhao et al., 2009; Song Y. Y. et al., 2015). Song et al. (2013) found that mycorrhizal colonization could initiate the JA signaling defense pathway in tomatoes and upregulate the expression of genes that synthesize LOX enzymes in response to caterpillar feeding. This could explain our result that LOX was significantly increased in mycorrhizal treatments. The LOX enzyme activity of Fm + Lm was approximately double that of the non-inoculated Lm treatment. Thus, despite the low levels of colonization in our experiments, we could still detect significant impacts of AMF on insect attack (Bernaola et al., 2018). However, the activities of PAL and β-1,3-glucanase decreased after AMF inoculation and grasshopper feeding did not notably increase these plant enzyme activities, suggesting that *E. nutans* may be predominantly protected against adverse external factors through the JA pathway and that the SA pathway does not play a dominant role. Previous studies found that once the JA defense pathway was up-regulated, the SA pathway was inhibited (Song Y. Y. et al., 2015; Schoenherr et al., 2019).

Previous research has also demonstrated that AMF could change the concentration and composition of VOCs (Sharma et al., 2017; Meier and Hunter, 2018), which alter plant attractiveness to insect behavior (Babikova et al., 2014; Meier and Hunter, 2019). The genus *Glomus* can be seen as a potent AMF in imparting biotic stress tolerance in a wide range of plants that are eaten by herbivores (Dowarah et al., 2021). Our results, in addition to findings from previous studies, clearly show that some of the genus *Glomus* fungi could induce plants to produce large amounts of volatile compounds, and these VOCs included terpenes, alcohols, esters, and small amounts of alkanes (Roger et al., 2013). In the present study, insect feeding led *E. nutans* to produce more terpenoids such as 2,4-Dimethylacetophenone and 2,5-Dimethylacetophenon, which have been shown to deter herbivores (Sharma et al., 2017). Ethyl acetate was only detected in the non-inoculated treatment, with low relative concentrations of other VOCs. The Fm + Lm treatment produced a greater variety of VOCs and metabolites with insect-repelling properties. AMF-inoculated plants increased the relative concentration of benzenic compounds compared to non-AMF plants. Benzenic compounds are the main components of various essential oils and are involved in plant reproduction and defense (Dudareva et al., 2004; Velásquez et al., 2020a). D-limonene and 1,3-diethylbenzene could be produced in other insect-infested plants and it has an important role in improving plant defenses and the repellence of insects (Agut et al., 2015; Kigathi et al., 2019; Mitra et al., 2021). p-Xylene mainly acts as an attractant to natural enemies of the feeding insect (Li et al., 2022). Taken together, these results suggest that AMF *F. mosseae* colonization can improve the chemical defense of *E. nutans*.

**FIGURE 4:**
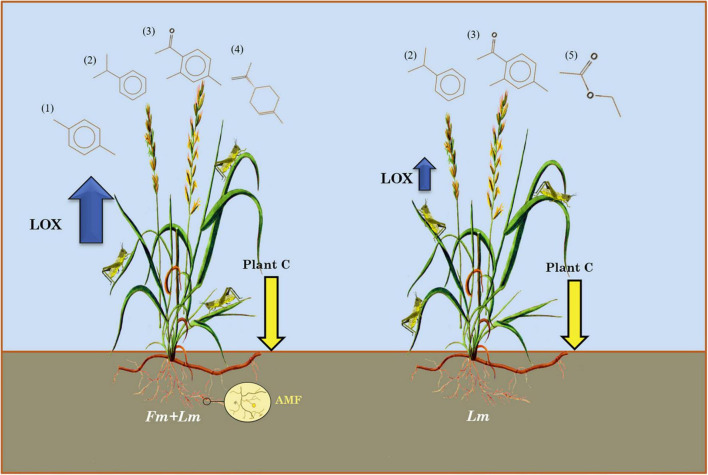
Diagrammatic representation of the various means of *E. nutans* defense response induction by AMF. (1) p-Xylene, (2) Cumene, (3) 2,4-dimethylacetophenone, (4) D-Limonene, and (5) ethyl acetate.

In conclusion, inoculation of *E. nutans* with *F. mosseae* improved the uptake of nutrients and induced resistance to grasshopper attack. AMF promote the expression of defense-related enzymes and the types of insect-resistant VOCs increased (Figure 4). The JA pathway was also found to be the main insect resistance pathway in *E. nutans*, with mycorrhizal colonization further inducing to strength the defense response. Furthermore, insect feeding could reduce the nutrient content of plants. Thus, the initiation of defense by common symbiotic organisms may be an important evolutionary strategy for plant defense against herbivores and other biotic stresses. Nevertheless, additional experiments are needed to assess whether *E. nutans* under AMF colonization in the field will initiate the same defense pathways and produce similar VOCs. This will help to clarify the mode and mechanism of AMF-grasshopper interactions and analyze the complex relationship of AMF-plant-herbivore interaction.

## Data Availability Statement

The original contributions presented in the study are included in the article/Supplementary Material, further inquiries can be directed to the corresponding author.

## Author Contributions

XS and KL conceived the study, supervised the writing, and revised the manuscript. WZ led the writing. LY and BH contributed sections to the manuscript. All authors read and approved the final submission.

## Conflict of Interest

The authors declare that the research was conducted in the absence of any commercial or financial relationships that could be construed as a potential conflict of interest.

## Publisher’s Note

All claims expressed in this article are solely those of the authors and do not necessarily represent those of their affiliated organizations, or those of the publisher, the editors and the reviewers. Any product that may be evaluated in this article, or claim that may be made by its manufacturer, is not guaranteed or endorsed by the publisher.
