# Supplementary material for: Mycorrhizal Inoculation Enhances Nutrient Absorption and Induces Insect-Resistant Defense of Elymus nutans
Source: Front Plant Sci. 2022 May 31;13:898969. doi: 10.3389/fpls.2022.898969 (PMC9194685; doi:10.3389/fpls.2022.898969)
Supplement: Supplementary file 1 [file Data_Sheet_1.docx]

Fig. S1 *E. nutants* of each treatment after 24 hours of grasshoppers feeding.


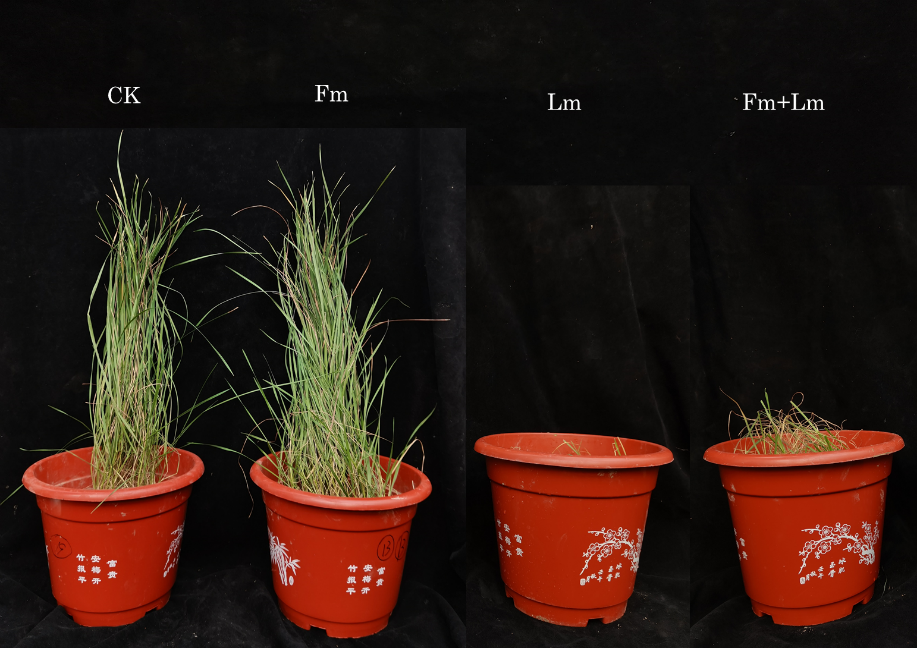


Table S1 Nutrient content of each plant under different treatments.

| Treatment | Total nitrogen  (g·plant^-1^) | Total phosphorus  (g·plant^-1^) | Total carbon  (g·plant^-1^) |
| --- | --- | --- | --- |
| CK | 0.00318±0.00022b | 0.00027±0.00002b | 0.08602±0.00630b |
| Fm | 0.00666±0.00042a | 0.00060±0.00004a | 0.16102±0.00928a |
| Lm | 0.00145±0.00004c | 0.00011±0.00001c | 0.04409±0.00074c |
| Fm+Lm | 0.00319±0.00031b | 0.00024±0.00002b | 0.08412±0.00767b |
